# Prognostic, Diagnostic, and Clinicopathological Significance of Circular RNAs in Pancreatic Cancer: A Systematic Review and Meta-Analysis

**DOI:** 10.3390/cancers14246187

**Published:** 2022-12-14

**Authors:** Jiajia Li, Ziping Ye, Xiaolin Hu, Sicong Hou, Qinglei Hang

**Affiliations:** 1 Department of Gastroenterology, The Affiliated Hospital of Yangzhou University, Yangzhou 225009, China; 2 Department of Gastroenterology, The First Affiliated Hospital of Nanjing Medical University, Nanjing 210029, China; 3 Department of Clinical Medicine, Medical College, Yangzhou University, Yangzhou 225001, China; 4 Department of Laboratory Medicine, Institute of Translational Medicine, Medical College, Yangzhou University, Yangzhou 225001, China; 5 Jiangsu Key Laboratory of Integrated Traditional Chinese and Western Medicine for Prevention and Treatment of Senile Diseases, Yangzhou 225001, China; 6 Jiangsu Key Laboratory of Experimental & Translational Noncoding RNA Research, Yangzhou University, Yangzhou 225001, China; 7 Jiangsu Key Laboratory of Zoonosis, Jiangsu Co-Innovation Center for Prevention and Control of Important Animal Infectious Diseases and Zoonoses, Yangzhou University, Yangzhou 225009, China; 8 Department of Experimental Radiation Oncology, The University of Texas MD Anderson Cancer Center, Houston, TX 77030, USA

**Keywords:** prognosis, diagnosis, clinicopathological characteristics, circular RNAs, pancreatic cancer, meta-analysis

## Abstract

**Simple Summary:**

Growing evidence shows that circRNAs are closely associated with the clinicopathological characteristics of pancreatic cancer (PC) patients and might facilitate the early diagnosis and prediction of prognosis after surgery. In this study, we reviewed a total of 48 studies to examine the clinical value of circRNAs in PC. We found that differentially expressed circRNAs are significantly correlated with the prognosis and clinicopathological features of PC patients and could assist the diagnosis, suggesting circRNAs as potent biomarkers in PC.

**Abstract:**

Pancreatic cancer (PC) is a highly aggressive malignant tumor with a high mortality rate. It is urgent to find optimal molecular targets for the early diagnosis and treatment of PC. Here, we aimed to systematically analyze the prognostic, diagnostic, and clinicopathological significance of circular RNAs (circRNAs) in PC. Relevant studies were screened through PubMed, Web of Science, and other databases. The prognostic value of PC-associated circRNAs was assessed using the composite hazard ratio (HR), the diagnostic performance was assessed using the area under the summary receiver operator characteristic (SROC) curve (AUC), and the correlation with clinicopathological characteristics using the composite odds ratio (OR) was explored. In our study, 48 studies were included: 34 for prognosis, 11 for diagnosis, and 30 for correlation with clinicopathological characteristics. For prognosis, upregulated circRNAs were associated with poorer overall survival (OS) (HR = 2.02) and disease-free survival/progression-free survival (HR = 1.84) while downregulated circRNAs were associated with longer OS (HR = 0.55). Notably, the combination of circRNAs, including hsa_circ_0064288, hsa_circ_0000234, hsa_circ_0004680, hsa_circ_0071036, hsa_circ_0000677, and hsa_circ_0001460, was associated with worse OS (HR = 2.35). For diagnosis, the AUC was 0.83, and the pooled sensitivity and specificity were 0.79 and 0.73, respectively. For clinicopathologic characteristics, upregulated circRNAs were associated with poorer tumor differentiation, more nerve and vascular invasion, higher T stage, lymphatic metastasis, distant metastasis, advanced TNM stage, and higher preoperative CA19-9 level. In contrast, downregulated circRNAs were negatively associated with PC differentiation and lymphatic metastasis. Overall, our results showed that circRNAs are closely related to the prognosis and clinicopathological characteristics of PC patients and could be utilized for early diagnosis; thus, they are promising biomarkers for clinical application in PC.

## 1. Introduction

Pancreatic cancer (PC) is a highly malignant solid tumor with morbidity almost equal to fatality. Surgery is currently the only possible cure for PC, but less than 20% of patients are able to undergo surgery when their cancer is diagnosed [1,2]. This may be attributed to the fact that PC has an insidious onset and tends to metastasize at an early stage. As a result, the 5-year survival rate of PC patients is still lower than 8% despite the significant progress in surgical techniques, radiotherapy, chemotherapy, and other adjuvant therapies [3,4]. At present, with the introduction of the concept of precise and personalized treatment, early screening, early diagnosis, and early treatment, as well as the search for biological targets for postoperative adjuvant therapy, have become the key to extending the survival time of PC patients [5,6].

Circular RNAs (circRNAs), once considered to be the product of molecular fragments or RNA mis-splicing, originate from precursor mRNAs with low expression abundance [7]. Generated from the 5′ splice donor site and 3′ splice acceptor site of precursor mRNAs (pre-mRNAs), circRNAs are covalently linked in reverse order. Because circRNAs lack the 5’ cap and 3’ tail structures and contain covalent continuous closed-loop structures, they are able to resist exonuclease action with high stability [8]. The formation mechanism of circRNAs mainly includes the formation of lasso structures by exon skipping, the removal of introns by internal splicing, and finally, the formation of ring structures by complementary base pairing in the intron repeat region on both sides of exons, which has not yet been fully clarified [9]. Lariat-driven circularization and intron-pairing-driven circularization are common models of circRNA formation. Additionally, some RNA binding proteins (RBPs) might act as regulatory activators or inhibitors in circRNA biogenesis [10,11,12]. By far, there are four main categories of known circRNAs: exonic circRNAs, exon-intron circRNAs, circular intronic RNAs, and other types of circRNAs [13,14]. By competing with conventional splicing, acting as transcriptional modulators, serving as translation templates of proteins, functioning as competing endogenous RNAs (ceRNAs), and binding to proteins, circRNAs participate in multiple physiological and pathological processes at the transcriptional or posttranscriptional level [15]. Explicitly expressed in different cell types, tissues, and developmental stages, circRNAs have been reported to contribute to various diseases, such as cardiovascular diseases, diabetes, and neurological disorders [16,17]. Importantly, with the development of sequencing and bioinformatics technology, circRNAs have been shown to play essential roles in the occurrence, development, metastasis, drug resistance, and other biological behaviors of various tumors [18]. A large number of differentially expressed circRNAs in PC have also been verified by microarray studies [19]. In recent years, the role of circRNAs in PC has been gradually explored, and circRNAs have been proved to be involved in the regulation of PC progression through a variety of pathways. For example, circFOXK2 promotes PC growth and metastasis by absorbing miR-942 and forming a complex with Ybx1/hnRNPK [20]. Additionally, there is growing evidence showing that circRNAs are closely associated with the clinicopathological characteristics of PC patients, and circRNAs are also helpful in the early diagnosis and determination of patient survival after surgery. Therefore, this meta-analysis was designed to investigate the comprehensive value of circRNAs in the prognosis, diagnosis, and clinicopathological significance of PC.

## 2. Materials and Methods

### 2.1. Literature Search Strategy

Electronic databases, including PubMed, Web of Science, Embase, and the Cochrane Library, were searched for relevant studies using both Medical Subject Heading (MeSH) terms and free words, including the following: “Pancreatic Cancer”, “Cancer of Pancreas”, “Pancreas Cancer”, “pancreatic ductal adenocarcinoma”, “Carcinoma, Pancreatic Duct”, “circular RNA”, “circRNA”, and “RNA, Circular”. References of the publications were also screened to prevent the omission of any valuable data. All articles were published in English, and the latest article was updated in June 2022. This study was conducted following the Preferred Reporting Items for Systematic Reviews and Meta-Analyses (PRISMA) and was registered in the International Prospective Register of Systematic Reviews (PROSPERO; registration number 42022303048). The PRISMA checklist is shown in Table S1.

### 2.2. Inclusion Criteria and Exclusion Criteria

Two independent authors (Jiajia Li and Ziping Ye) screened the publications carefully to identify eligible studies. For inclusion, studies had to meet the following inclusion criteria: (1) a case-control study design was used; (2) pathological confirmation of PC diagnosis was reported for all patients; and (3) estimates (directly or indirectly extracted) of prognostic, the diagnostic accuracy of circRNAs in PC, or relationship of circRNAs with PC clinicopathological characteristics were provided. Exclusion criteria: (1) not related to PC or circRNAs; (2) letters, reviews, or duplicated publications; (3) studies lacking sufficient data for our analysis. In cases of discordance, a consensus was reached through discussion with another author (Sicong Hou).

### 2.3. Data Extraction and Quality Assessment

To ensure accuracy, two investigators extracted data from eligible studies independently. The following data was recorded in a standard form: name of the first author, name of the circRNA, year of publication, country, expression of circRNA, cut-off value, sample size, detected sample, detection method, follow-up time (months), survival outcomes including overall survival (OS), disease-free survival (DFS) or progression-free survival (PFS), survival analysis method (univariate or multivariate), and hazard ratio (HR) with 95% confidence interval (CI). Regarding the diagnostic value, the following data was collected: sample size, detected sample, area under the receiver operating characteristic (ROC) curve (AUC), sensitivity, specificity, number of true positive (TP), false positive (FP), true negative (TN), false negative (FN), positive likelihood ratio (PLR), negative likelihood ratio (NLR), and diagnostic odds ratio (DOR). Additionally, clinicopathological characteristics, including gender, age, CA19-9 level, location, nerve invasion, vascular invasion, duodenal invasion, differentiation, tumor size, T stage, lymphatic metastasis, distant metastasis, and TNM stage, were also recorded.

The Quality Assessment for Studies of Diagnostic Accuracy II (QUADAS II) checklist was used for the quality assessment of diagnostic studies, and the Newcastle-Ottawa score (NOS) quality assessment system was applied for prognostic studies [21,22]. Studies with a QUADAS II score ≥ 4 or a NOS score ≥ 7 were considered of high quality.

### 2.4. Statistical Analysis

This meta-analysis was carried out using Stata software (version 15.1). The rating results were visualized with the Review Manager software (version 5.4). Pooled HRs or odds ratios (ORs) with 95% CIs were calculated to evaluate the relationship between circRNAs and the prognosis as well as clinicopathological characteristics of PC. Engauge Digitizer software and GetData Graph Digitizer software were applied to extract data from the Kaplan-Meier curve or ROC curve when necessary [23]. A summary receiver operator characteristic (SROC) curve was generated to explore the pooled diagnostic value of circRNAs in PC. Fagan’s nomogram and scatter plots of PLR and NLR were used to assess their clinical application value. We evaluated heterogeneity using two methods (Cochran’s Q statistic and I^2^) under homozygous and recessive models. A *p*-value > 0.1 and I^2^ < 50% indicate that the heterogeneity is not statistically significant, and the fixed effects model should be chosen [24]. Otherwise, the random effects model would be more appropriate [25]. Additionally, a bivariate boxplot was utilized to analyze the heterogeneity of the diagnostic experiments. A sensitivity analysis was performed to assess the stability of studies on the pooled HRs by omitting one single study each time and calculating the outcome again. Publication bias was estimated with Deeks’ funnel plots, Begg’s funnel plots, and Egger’s test. A *p* < 0.05 indicates significant publication bias.

## 3. Results

### 3.1. Selection of Literatures

**Figure 1:**
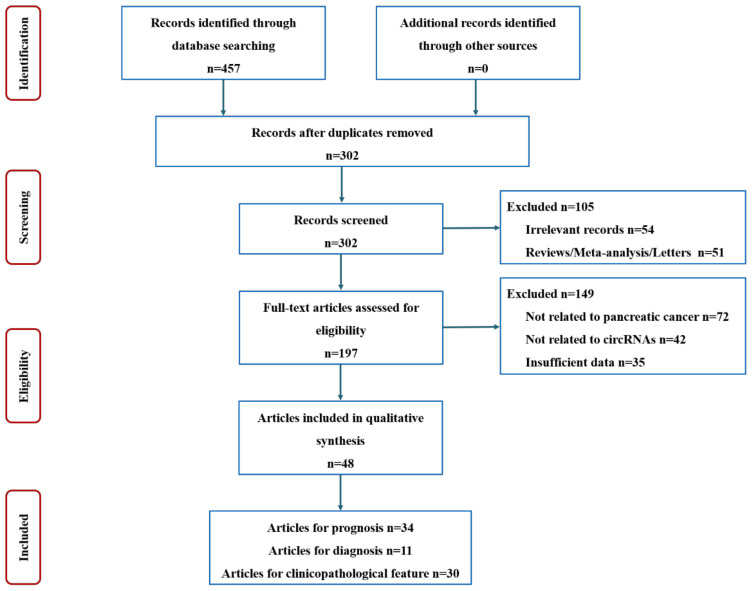
Flow diagram of the study selection process.

The procedure of literature selection is summarized in Figure 1. The search strategy yielded 457 articles. After the removal of 155 duplicates, 302 citations remained. Then, based on the title or abstract, 105 citations were excluded, and the remaining 197 articles were further scanned. Another 149 studies were removed for not being related to PC or circRNAs, or not providing sufficient data. Ultimately, a total of 48 papers were included in our meta-analysis, including 34 on prognosis [26,27,28,29,30,31,32,33,34,35,36,37,38,39,40,41,42,43,44,45,46,47,48,49,50,51,52,53,54,55,56,57,58,59], 11 on diagnosis [29,30,31,48,55,56,60,61,62,63,64], and 30 on relationship with clinicopathological features [28,29,30,31,32,33,34,35,39,41,42,43,44,45,46,48,50,51,55,56,58,59,62,64,65,66,67,68,69,70]. The characteristics of each study are shown in Table 1 and Table 2.

### 3.2. Association between Expression of circRNAs and Prognosis in PC

According to the literature, a total of 34 circRNAs were associated with PC prognosis, of which 28 were upregulated, and the remaining 6 were downregulated (Table 1). All the studies were published from 2018 to 2022, with follow-up periods ranging from 20 to 80 months. The sample size ranged from 26 to 209, and the median was adopted as the cut-off value for circRNA expression in most cases. Except for the studies by Xie, H.R. et al., Rong, Z.Y. et al., and Han, X. et al., which used the tissue microarray (TMA) RNA-in situ hybridization (ISH)/fluorescence in situ hybridization (FISH) methods for detection, the expression of circRNAs was detected by quantitative real-time polymerase chain reaction (qRT-PCR). The specimens were obtained from tumor tissue or plasma, and all PC diagnosis was pathologically confirmed. The NOS quality assessment system was used to evaluate the quality of the included studies, and all of the studies achieved a score ≥ 7 (Figure S1).

**Figure 2:**
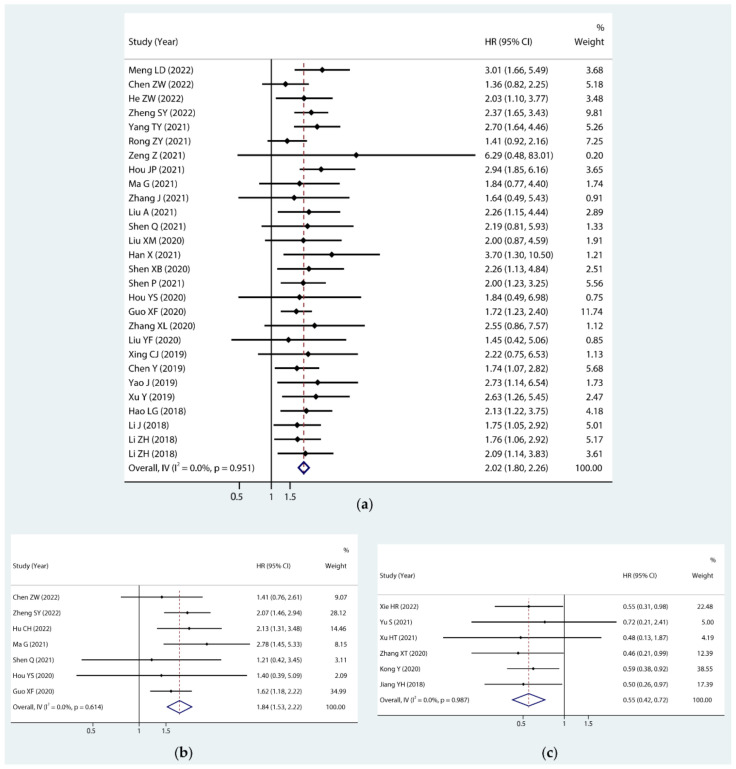
Forest plots of the OS (**a**), DFS/PFS (**b**) for upregulated circRNAs, and OS (**c**) for downregulated circRNAs.

As shown in Figure 2, PC-associated upregulated circRNAs were correlated to a poorer OS (HR = 2.02, 95% CI: 1.80–2.26) and poorer DFS/PFS (HR = 1.84, 95% CI: 1.53–2.22). In contrast, patients with downregulated circRNAs had a better OS (HR = 0.55, 95% CI: 0.42–0.72). The statistical results showed that the circRNAs had no significant heterogeneity(I^2^ = 0.0%); thus, a fixed effects model was used for the analysis.

### 3.3. Association between Expression of circRNAs and Diagnosis in PC

**Figure 3:**
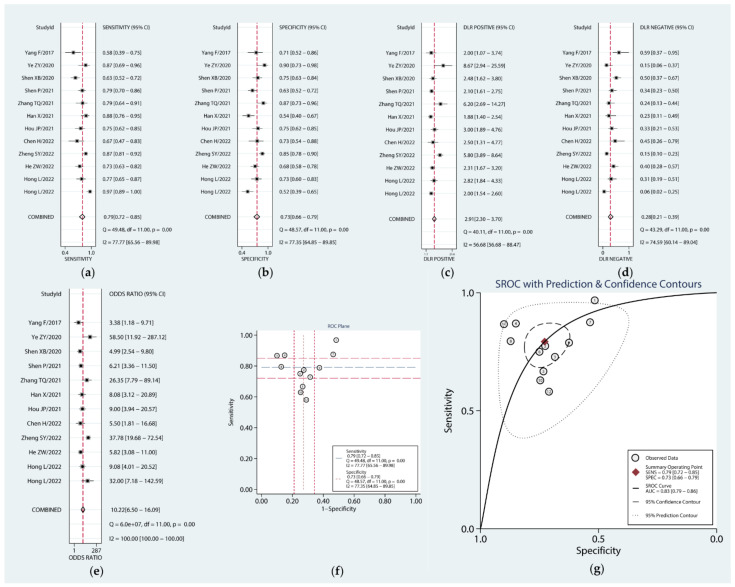
Forest plots of the combined sensitivity (**a**), specificity (**b**), PLR (**c**), NLR (**d**), DOR (**e**), ROC plane (**f**), and SROC curve (**g**) to illustrate the diagnostic value of circRNAs in pancreatic cancer.

Twelve studies were eligible for our diagnostic meta-analysis, and the QUADAS II scores showed that all of the studies were of high quality (Figure S2). The forest plot (Figure 3a–e) showed that the combined sensitivity, specificity, PLR, NLR, and DOR were 0.79 (95% CI: 0.72–0.85), 0.73 (95% CI: 0.66–0.79), 2.91 (95% CI: 2.30–3.70), 0.28 (95% CI: 0.21–0.39), and 10.22 (95% CI: 6.50–16.09), respectively. The ROC plane diagram did not show a typical shoulder–arm distribution, suggesting no threshold effect (Figure 3f). Then, we plotted the SROC curve, which yielded an AUC of 0.83 (95% CI: 0.79–0.86) (Figure 3g). These results suggest that circRNAs can be utilized as ideal biomarkers for diagnosing PC.

**Figure 4:**
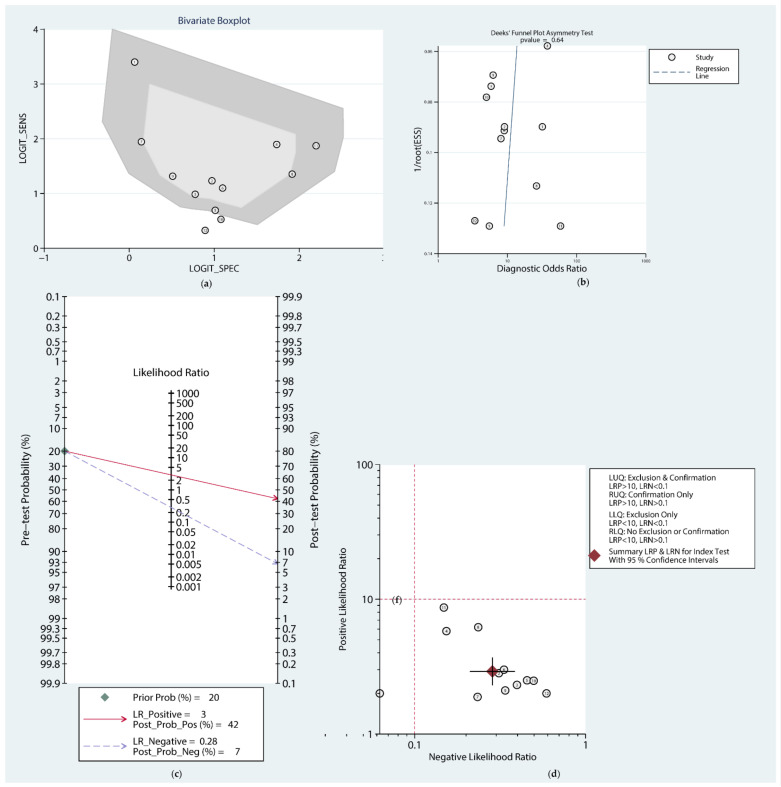
Bivariate boxplot (**a**), Deeks’ funnel plot (**b**), Fagan’s nomogram (**c**), and Scatter plot of PLR and NLR (**d**) to illustrate the diagnostic accuracy of circRNAs in pancreatic cancer. “LRP” and “LRN” in the text within Figure 4d refer to the labels of *Y*-axis (Positive Likelihood Ratio, PLR) and *X*-axis (Negative Likelihood Ratio, NLR), respectively.

As shown in Figure 4a, the bivariate boxplot did not show any significantly heterogeneous data, and no significant publication bias was detected (Figure 4b). Although the bivariate boxplot did not show significantly heterogeneous data (Figure 4a), the overall sensitivity, specificity, PLR, NLR, and DOR were all highly heterogeneous (Figure 3). Therefore, a subgroup analysis and meta regression based on the detected sample, case size, and publication year were conducted (Tables S2 and S3). It turned out that no significant correlation between the covariates and the DOR was detected in the univariate meta-regression analysis. To further evaluate the clinical application capability of circRNAs, we constructed Fagan’s nomogram (Figure 4c) and a scattered plot of PLR and NLR (Figure 4d). It was found that with 20% as a prior probability, the PLR postposterior probability increased to 42% (PLR = 3) while the NLR postposterior probability decreased to 7% (NLR = 0.28). Therefore, circRNAs can be used as feasible and reliable diagnostic markers for PC.

### 3.4. Association between Expression of circRNAs and Clinicopathological Characteristics in PC

Table 3 displays the correlation between circRNAs and the clinicopathological characteristics of PC. Our analysis demonstrated that upregulated circRNAs were associated with higher preoperative CA19-9 levels (OR = 2.082, 95% CI: 1.462–2.966), more nerve and vascular invasion (OR = 1.637, 95% CI: 1.163–2.304 and OR = 2.006, 95% CI: 1.451–2.773), poorer tumor differentiation (OR = 1.914, 95% CI: 1.519–2.410), larger tumor size (OR = 2.442, 95% CI: 1.580–3.774), higher T stage (OR = 1.685, 95% CI: 1.317–2.156), lymphatic metastasis (OR = 3.404, 95% CI: 2.706–4.283), distant metastasis (OR = 4.394, 95% CI: 2.564–7.532), and advanced TNM stage (OR = 3.282, 95% CI: 2.463–4.374). There was no correlation between circRNA expression and other indicators, including gender, age, location, and duodenal invasion (Table 3). Fewer studies focused on downregulated circRNAs in PC, and downregulated circRNAs were associated with better differentiation (OR = 0.367, 95% CI: 0.220–0.611) and less lymphatic metastasis (OR = 0.121, 95% CI: 0.070–0.209). It is worth noting that the I^2^ index showed high heterogeneity; therefore, more studies need to be included for subgroup analysis in future research.

### 3.5. Sensitivity Analysis and Publication Bias

To assess the sensitivity of our meta-analysis, one single study was omitted from the analysis each time to calculate the pooled HRs or ORs again (Figures S3–S5). The detailed data suggest that the results are stable.

Possible publication bias was assessed using Begg’s funnel plot and Egger’s test (Figures S6–S9), and there was no significant publication bias in the results. Moreover, Deeks’ funnel plot asymmetry test (*p* = 0.64) suggested that no significant publication bias in the diagnostic analysis (Figure 4b).

### 3.6. Clinical Value Evaluation of Combined circRNAs

**Figure 5:**
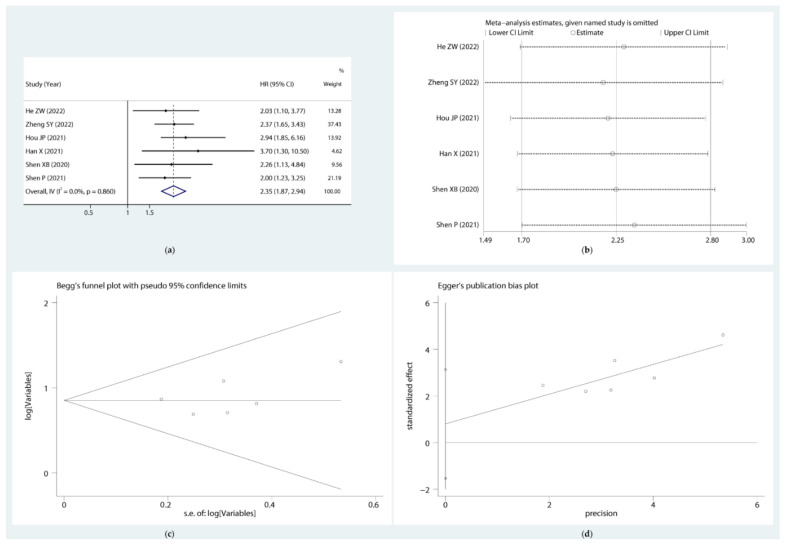
Forest plot of the OS (**a**), sensitivity analysis (**b**), and publication bias judged by Begg’s (**c**) and Egger’s (**d**) funnel plots of combined circRNAs in pancreatic cancer.

**Figure 6:**
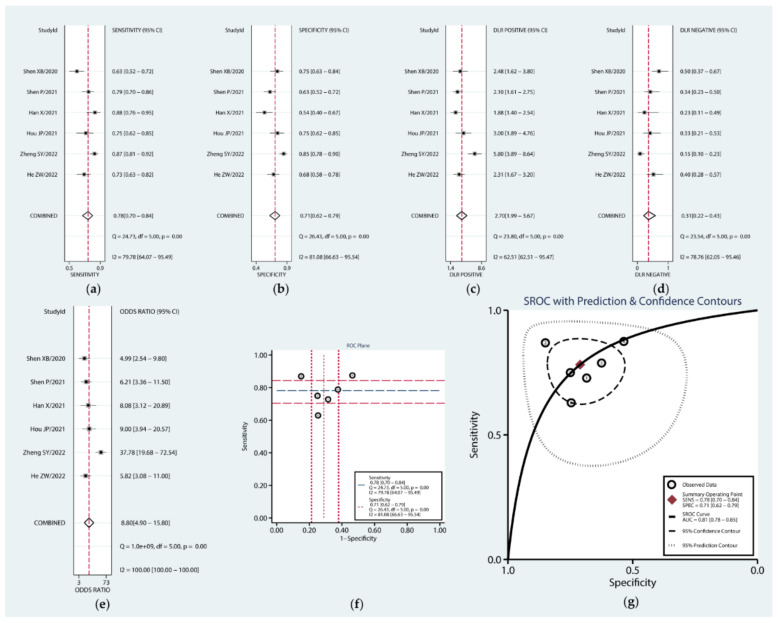
Forest plots of the sensitivity (**a**), specificity (**b**), PLR (**c**), NLR (**d**), DOR (**e**), ROC plane (**f**), and SROC curve (**g**) of combined circRNAs in pancreatic cancer.

Among the 12 circRNAs with diagnostic value, we screened out 6 circRNAs with prognostic data to detect their combined prognostic and diagnostic value. They were hsa_circ_0064288, hsa_circ_0000234, hsa_circ_0004680, hsa_circ_0071036, hsa_circ_0000677, and hsa_circ_0001460, which were all higher in PC tissues than in para-cancer tissues. The results showed that combined circRNAs were significantly associated with poor OS (HR = 2.35, 95% CI: 1.87–2.94) with no significant bias (Figure 5). Moreover, the forest plot (Figure 6a,b) showed that the combined sensitivity and specificity of these 6 circRNAs was 0.78 (95% CI: 0.70–0.84) and 0.71 (95% CI: 0.62–0.79), respectively, and the SROC curve yielded an AUC of 0.81 (95% CI: 0.78–0.85) (Figure 6g).

## 4. Discussion

PC is known to be the most aggressive tumor of the digestive system, and unlike the steady increase of survival rates observed in other cancers, the 5-year-survival rate of PC has barely changed in recent years [71,72]. The results of individualized therapy suggest that targeted therapy is feasible to improve the prognosis of PC patients. Therefore, identifying novel targets during the development and progression of PC is a prerequisite for improving the prognosis [73,74]. Non-coding RNAs (ncRNAs) are generated from human genomes without the ability to encode proteins. They are contained in cells and exosomes of body fluids, and they have broad application prospects in disease diagnosis. According to the length and shape of ncRNAs, they are generally classified as microRNAs (miRNAs), piwi-acting RNAs (piRNAs), long non-coding RNAs (lncRNAs), small nucleolar RNAs (snoRNAs), and circRNAs [75]. So far, numerous ncRNAs have been identified as oncogenic drivers and contribute to tumor proliferation or metastasis among a variety of cancers [76]. In PC, it has been reported that miRNA holds potential as biomarkers, prognostic markers, and clinical targets [77]. Another review confirmed the ability of lncRNAs to improve the early diagnosis, prognostic prediction, and personalized treatments of patients with PC [78]. However, the role of circRNAs in PC diagnosis and prognosis is still controversial. CircRNAs are closed circular RNA molecules that have drawn more and more attention in recent years [79]. They are not easy to hydrolyze with RNA enzymes and are highly abundant in expression [80]. In addition, circRNAs are highly conserved during the evolution in different species, and their expression profiles are tissue- and developmental stage-specific [81]. At the cellular level, a large number of circRNAs exist in the cytoplasm of eukaryotic cells. Most of these circRNAs act as competitive endogenous RNAs, bind to RNA-binding proteins, regulate gene transcription, and even encode proteins, which are the molecular basis for their biological functions [82,83]. Considering the wide distribution of circRNAs in tissues, serum, and urine, their expression levels have consistently been correlated with clinicopathological features of various tumors, suggesting their potential as biomarkers for cancer diagnosis and prognosis [84,85]. With the emergence of new research toward identifying circRNAs as diagnostic biomarkers and therapeutic targets for PC, we aimed to explore the comprehensive value of circRNAs in the prognosis, diagnosis, and clinicopathological significance of PC.

By reviewing PubMed, Web of Science, and other databases and performing quality assessments, we screened and filtered out 48 studies on PC-related circRNAs. For the relationship between circRNAs and PC prognosis, we included 34 studies with a cumulative total of 3524 patients. The results showed that upregulated circRNAs were positively correlated with the OS/DFS/PFS of patients. Although fewer studies focused on downregulated circRNAs, 3 studies showed that they are negatively associated with the OS of PC patients. To our knowledge, this is the first meta-analysis investigating the association of circRNAs with the prognosis, diagnosis, and clinicopathological characteristics in PC patients. Several previous articles have investigated the relationship between circRNAs and other tumors, including lung cancer, osteosarcoma, squamous cell carcinoma, esophageal squamous cell carcinoma, etc. [86,87,88]. Wang et al. have shown that the elevated expression of oncogenic circRNAs could predict poor survival outcomes (HR = 2.430 for OS; HR = 2.228 for DFS) in lung cancer, and similar results were obtained by Nie et al. in esophageal squamous cell carcinoma (HR = 2.25 for OS) [86,88]. The pooled results produced in this study are close to these previously reported cancers, indicating that circRNAs could also facilitate the prognosis estimation in PC. So far, due to the lack of a unified standard, the quantification of each circRNA is not precise enough. Additionally, there is a lack of more abundant evaluation indicators, such as the OS/DFS/PFS of chemotherapy-sensitive patients, so more refined studies must be carried out. Additionally, regarding the differences in circRNA origin and detection method, it would be better if subgroup analysis could be executed based on circRNA origin and detection method. However, as shown in Table 1, a total of 34 circRNAs were included for exploring their prognostic value in PC. Still, among them, only one circRNA (hsa_circ_0036627) was detected in plasma, which is inadequate for statistical analysis. There is a similar trend with the detection method. Among the 28 upregulated circRNAs, only 2 studies by Rong ZY et al. and Han X et al. used the tissue microarray (TMA) RNA-fluorescence in situ hybridization (FISH) method rather than the quantitative real-time polymerase chain reaction (qRT-PCR) for circRNA detection. Therefore, a data split based on origin and detection method is not feasible here. To further validate these results, large-scale follow-up studies are needed. In studies researching PC clinicopathologic features, upregulated circRNAs were significantly correlated with tumor differentiation, vascular invasion, and TNM stage, suggesting an indispensable role in promoting the development and progression of PC. In contrast, downregulated circRNAs were associated with better differentiation and lower TNM stage.

Regarding the diagnostic value of circRNAs in PC, we included 11 diagnostic studies with a total of 824 cases and 798 controls. The combined sensitivity, specificity, and AUC for diagnosis were 0.79, 0.73, and 0.83, implying that circRNAs were upregulated in 83% of PC patients compared to controls. At the same time, the overall DOR for circRNA diagnosis was 10.22, PLR was 2.91, and NLR was 0.28, suggesting that patients with high circRNA expression had a 2.91-fold higher risk of PC than the general population. In contrast, the risk of those with no differential expression was only 0.32 folds. Fagan’s nomogram was used to analyze the clinical value of circRNAs, and the results showed a significant increase in PLR and a significant decrease in NLR. These results were consistent with previous studies researching circRNAs’ diagnostic value in other cancers [89,90]. Moreover, we detected the prognostic value of the combined circRNAs composed of hsa_circ_0064288, hsa_circ_0000234, hsa_circ_0004680, hsa_circ_0071036, hsa_circ_0000677, and hsa_circ_0001460. The results showed that they demonstrated a higher prognostic value for PC patients. Compared with CA 19-9, the most classical and widely-used indicator of PC, which was reported to possess a sensitivity and specificity of 78.2% and 82.8%, it seems that circRNAs did not show much superiority over CA 19-9 on PC diagnosis [91]. In spite of this, circRNAs as biomarkers in PC still have the following advantages. First, some circRNAs, including hsa_circ_0004680 and hsa_circ_0001666, showed better discriminatory power in detecting PC patients from healthy controls, especially in PC with serum CA19-9 < 37 U/mL, suggesting that circRNAs might serve as a preferable biomarker for detecting PC [48,63]. Second, numerous reports have revealed the association between circRNA expression and chemotherapy resistance during PC treatment, implicating that the expression of circRNAs might have the potential to facilitate the selection of treatment options [92,93]. Lastly, considering that circRNAs play indispensable roles in cancer pathogenesis, it is promising to design potential diagnostic and therapeutic strategies targeting circRNAs to gain control of PC. Thus far, however, no treatment based on circRNAs has yet been approved in the clinic. With the continued emergence of more gratifying investigations, we believe that more characteristic circRNAs with diagnostic and therapeutic potency in PC will be identified.

To the best of our knowledge, this research is the first meta-analysis investigating the comprehensive role of circRNAs in PC. However, it should be noted that there were several limitations in this meta-analysis. First, the number of studies included was relatively small, which makes it infeasible to perform subgroup analysis based on circRNA origin or detection method. To further validate these results, large-scale follow-up studies are needed. Second, all the studies were performed in China, so more diverse populations should be studied in the future. Finally, the vast majority of the current studies were based on PC tissues, which means that more high-quality studies that use blood, which is easily accessible preoperative, are needed.

## 5. Conclusions

In conclusion, our study showed that the differentially expressed circRNAs in PC (especially the combined circRNAs- hsa_circ_0064288, hsa_circ_0000234, hsa_circ_0004680, hsa_circ_0071036, hsa_circ_0000677, and hsa_circ_0001460) are significantly correlated with the prognosis and clinicopathological characteristics of PC patients and could contribute to the diagnosis of PC. However, since this conclusion is based on a limited number of studies, more relevant research is needed to validate our results.

## Figures and Tables

**Table 1:** Characteristics of prognostic studies included in the meta-analysis.

Study	Circ	Year	Expression	Cut-Off	Sample Size	Detected Sample	Detection Method	Follow-Up Time (Months)	Survival Outcome	Survival Analysis	Variables		
											HR ^g^	95% CI ^h^	*p* Value
Meng, L.D.	0007905	2022	Up	Median	97	Tissue	qRT-PCR	80	OS ^b^	Multi ^e^	3.013	1.655–5.486	<0.001
Chen, Z.W.	0078297	2022	Up	Median	96	Tissue	qRT-PCR	80	PFS ^c^	Uni ^f^	1.41	0.76–2.61	0.019
Chen, Z.W.	0078297	2022	Up	Median	96	Tissue	qRT-PCR	80	OS	Uni	1.36	0.82–2.25	0.035
Xie, H.R.	0047744	2022	Down	N/A ^a^	69	Tissue	ISH-TMA	60	OS	Uni	0.55	0.31–0.98	0.0043
He, Z.W.	0064288	2022	Up	Median	92	Tissue	qRT-PCR	60	OS	Uni	2.03	1.1–3.77	0.0023
Zheng, S.Y.	0000234	2022	Up	Median	161	Tissue	qRT-PCR	80	OS	Multi	2.374	1.645–3.426	<0.001
Zheng, S.Y.	0000234	2022	Up	Median	161	Tissue	qRT-PCR	80	PFS	Multi	2.073	1.460–2.942	<0.001
Hu, C.H.	0002557	2022	Up	N/A	82	Tissue	qRT-PCR	20	PFS	Uni	2.13	1.31–3.48	<0.001
Yu, S.	0092367	2021	Down	N/A	40	Tissue	qRT-PCR	60	OS	Uni	0.72	0.21–2.41	0.007
Xu, H.T.	0013587	2021	Down	Median	30	Tissue	qRT-PCR	60	OS	Uni	0.48	0.13–1.87	0.005
Yang, T.Y.	0007444	2021	Up	Median	110	Tissue	qRT-PCR	80	OS	Multi	2.7	1.636–4.456	<0.001
Rong, Z.Y.	0007895	2021	Up	Fluorescence ratio	209	Tissue	FISH-TMA	70	OS	Uni	1.41	0.92–2.16	0.004
Zeng, Z.	ZNF91	2021	Up	Median	40	Tissue	qRT-PCR	20	OS	Uni	6.29	0.48–83.01	<0.05
Hou, J.P.	0004680	2021	Up	Median	30	Tissue	qRT-PCR	60	OS	Multi	2.94	1.85–6.16	0.007
Ma, G.	0005105	2021	Up	N/A	75	Tissue	qRT-PCR	60	OS	Uni	1.84	0.77–4.4	0.00066
Ma, G.	0005105	2021	Up	N/A	75	Tissue	qRT-PCR	60	DFS ^d^	Uni	2.78	1.45–5.33	0.00021
Zhang, J.	0066147	2021	Up	Mean	45	Tissue	qRT-PCR	60	OS	Uni	1.64	0.49–5.43	0.0145
Liu, A.	03955	2021	Up	N/A	56	Tissue	qRT-PCR	80	OS	Uni	2.26	1.15–4.44	<0.001
Shen, Q.	0092314	2021	Up	Median	40	Tissue	qRT-PCR	60	OS	Uni	2.19	0.81–5.93	0.001
Shen, Q.	0092314	2021	Up	Median	40	Tissue	qRT-PCR	60	DFS	Uni	1.21	0.42–3.45	0.0231
Liu, X.M.	0001013	2020	Up	Median	80	Tissue	qRT-PCR	40	OS	Uni	2	0.87–4.59	<0.05
Han, X.	0071036	2021	Up	Fluorescence ratio	84	Tissue	FISH-TMA	48	OS	Multi	3.7	1.3–10.5	0.015
Shen, X.B.	0000677	2020	Up	Median	26	Tissue	qRT-PCR	60	OS	Multi	2.256	1.132–4.836	0.042
Shen, P.	0001460	2021	Up	Median	104	Tissue	qRT-PCR	75	OS	Multi	1.995	1.225–3.248	0.006
Hou, Y.S.	0005273	2020	Up	N/A	56	Tissue	qRT-PCR	80	OS	Uni	1.84	0.49–6.98	<0.05
Hou, Y.S.	0005273	2020	Up	N/A	56	Tissue	qRT-PCR	80	PFS	Uni	1.4	0.39–5.09	<0.05
Zhang, X.T.	0000979	2020	Down	Median	67	Tissue	qRT-PCR	60	OS	Uni	0.46	0.21–0.99	0.043
Guo, X.F.	0009065	2020	Up	Median	208	Tissue	qRT-PCR	60	OS	Multi	1.718	1.228–2.402	0.002
Guo, X.F.	0009065	2020	Up	Median	208	Tissue	qRT-PCR	60	DFS	Multi	1.62	1.183–2.217	0.003
Kong, Y.	0086375	2020	Down	Median	160	Tissue	qRT-PCR	60	OS	Multi	0.593	0.382–0.920	0.02
Kong, Y.	0086375	2020	Down	Median	160	Tissue	qRT-PCR	60	DFS	Multi	0.587	0.386–0.895	0.013
Zhang, X.L.	0001568	2020	Up	Median	83	Tissue	qRT-PCR	60	OS	Uni	2.55	0.86–7.57	<0.05
Liu, Y.F.	0000284	2020	Up	N/A	28	Tissue	qRT-PCR	60	OS	Uni	1.45	0.42–5.06	0.043
Xing, C.J.	ADAM9	2019	Up	N/A	58	Tissue	qRT-PCR	40	OS	Uni	2.22	0.75–6.53	0.001
Chen, Y.	ASH2L	2019	Up	Median	85	Tissue	qRT-PCR	50	OS	Multi	1.741	1.075–2.821	0.024
Yao, J.	LDLRAD3	2019	Up	N/A	38	Tissue	qRT-PCR	60	OS	Uni	2.73	1.14–6.54	0.0476
Xu, Y.	0030235	2019	Up	N/A	62	Tissue	qRT-PCR	60	OS	Multi	2.626	1.264–5.455	0.01
Hao, L.G.	0007534	2018	Up	Median	60	Tissue	qRT-PCR	60	OS	Multi	2.135	1.217–3.745	0.008
Li, J.	IARS	2018	Up	Median	50	Tissue	qRT-PCR	50	OS	Multi	1.749	1.047–2.924	0.033
Jiang, Y.H.	0001649	2018	Down	Mean	58	Tissue	qRT-PCR	60	OS	Multi	0.502	0.261–0.966	0.039
Li, Z.H.	0036627	2018	Up	Median	93	Tissue	qRT-PCR	50	OS	Multi	1.764	1.064–2.925	0.028
Li, Z.H.	0036627	2018	Up	Median	56	plasma	qRT-PCR	50	OS	Multi	2.093	1.143–3.834	0.017

a, N/A: not available; b, OS: overall survival; c, PFS: progression-free survival; d, DFS: disease-free survival; e, Multi: multivariate; f, Uni: univariate; g, HR: hazard ratio; h, 95% CI: 95% confidence interval.

**Table 2:** Characteristics of diagnostic studies included in the meta-analysis.

Study	Circ	Year	Expression	Sample Size		Detected Sample	Variables									
				Case	Control		AUC ^b^	Sen ^c^	Spe ^d^	TP ^e^	FP ^f^	TN ^g^	FN ^h^	PLR ^i^	NLR ^j^	DOR ^k^
Hong, L.	0001666	2022	Up	62	62	Blood ^a^	0.8062	0.9677	0.5161	60	30	32	2	1.9998	0.0626	31.9534
Hong, L.	0006220	2022	Up	62	62	Blood	0.7817	0.7742	0.7258	48	17	45	14	2.8235	0.3111	9.0757
He, Z.W.	0064288	2022	Up	92	92	Tissue	0.7278	0.73131	0.68071	67	29	63	25	2.2904	0.3947	5.8027
Zheng, S.Y.	0000234	2022	Up	161	161	Tissue	0.871	0.86746	0.73837	140	21	119	21	3.3156	0.1795	18.4709
Chen, H.	0074298	2022	Up	30	30	Tissue	0.676	0.667	0.733	20	8	22	10	2.4981	0.4543	5.4989
Hou, J.P.	0004680	2021	Up	60	60	Blood	0.8015	0.7581	0.7581	45	15	45	15	3.1339	0.3191	9.8216
Han, X.	0071036	2021	Up	56	56	Tissue	0.65	0.8837	0.53	49	26	30	7	1.8802	0.2194	8.5697
Zhang, T.Q.	0060055	2021	Up	39	39	Tissue	0.9093	0.7947	0.87	31	5	34	8	6.1131	0.2360	26.9030
Shen, P.	0001460	2021	Up	104	104	Tissue	0.7364	0.7885	0.625	82	39	65	22	2.1027	0.3384	6.2137
Shen, X.B.	0000677	2020	Up	97	71	Blood	0.716	0.6276	0.7429	61	18	53	36	2.4411	0.5013	4.8695
Ye, Z.Y.	0000069	2020	Up	30	30	Tissue	0.8944	0.8774	0.89	26	3	27	4	7.9764	0.1378	57.8839
Yang, F.	0006988	2017	Up	31	31	Blood	0.67	0.5738	0.7049	18	9	22	13	1.9444	0.6046	3.2160

a, Blood: peripheral blood; b, AUC: area under the curve; c, Sen: sensitivity; d, Spe: specificity; e, TP: true positive; f, FP: false positive; g, TN: true negative; h, FN: false negative; i, PLR: positive likelihood ratio; j, NLR: negative likelihood ratio; k, DOR: diagnostic odds ratio.

**Table 3:** Meta-analysis of correlation between upregulated circRNAs and clinicopathological features of pancreatic cancer.

**Upregulated circRNAs**	**No. of Studies**	**No. of Patients**	**Odds Ratio (95%CI)**	***p* Value**	**Heterogeneity**	**I^2^ (%)**
Gender (male/female)	27	2230	0.952 (0.800–1.133)	0.580	13.63	0
Age (≥60/<60 years)	14	982	0.911 (0.699–1.186)	0.487	10.16	0
CA 19-9 (+/−)	8	733	2.082 (1.462–2.966)	<0.001	11.63	39.8
Location (head/body & tail)	16	1266	1.039 (0.816–1.322)	0.670	12.12	0
Nerve invasion (+/−)	9	902	1.637 (1.163–2.304)	0.005	8.08	1.0
Vascular invasion (+/−)	11	783	2.006 (1.451–2.773)	<0.001	21.82	54.2
Duodenal invasion (+/−)	3	268	1.416 (0.691–2.900)	0.342	0.04	0
Differentiation (low/moderate & well)	16	1473	1.914 (1.519–2.410)	< 0.001	24.24	38.1
Tumor size (>4/≤4 cm)	6	406	2.442 (1.580–3.774)	< 0.001	10.65	53
T stage (T3–4/T1–2)	11	1030	1.685 (1.317–2.156)	< 0.001	20.33	50.8
Lymphatic metastasis (+/−)	20	1464	3.404 (2.706–4.283)	< 0.001	21.59	12
Distant metastasis (+/−)	6	434	4.394 (2.564–7.532)	< 0.001	1.33	0
TNM stage (III–IV/I–II)	13	1175	3.282 (2.463–4.374)	< 0.001	47.48	74.7
**Downregulated circRNAs**	**No. of Studies**	**No. of Patients**	**Odds Ratio (95%CI)**	***p* Value**	**Heterogeneity**	**I^2^ (%)**
Gender (male/female)	3	285	1.179 (0.735–1.889)	0.495	1.3	0
Differentiation (low/moderate & well)	3	285	0.367 (0.220–0.611)	< 0.001	26.32	92.5
Lymphatic metastasis (+/−)	3	285	0.121 (0.070–0.209)	< 0.001	12.37	83.8

## Supplementary Materials

The following supporting information can be downloaded at: https://www.mdpi.com/article/10.3390/cancers14246187/s1, Table S1: The PRISMA checklist; Table S2: Assessment of the diagnostic accuracy and heterogeneity in subgroup analysis; Table S3: Results of univariate meta-regression analysis of diagnostic odds ratio; Figure S1: Quality assessment by QUADAS II. (a) Each risk of bias item for each included study; (b) Each risk of bias item presented as percentages across all included studies; Figure S2: Quality assessment by NOS. (a) Each risk of bias item for each included study; (b) Each risk of bias item presented as percentages across all included studies; Figure S3: Sensitivity analysis of OS (a), DFS/PFS (b) for upregulated circRNAs and OS (c) for downregulated circRNAs in pancreatic cancer; Figure S4: Sensitivity analysis of upregulated circRNAs for CA19-9 (a), nerve invasion (b), vascular invasion(c), differentiation (d), tumor size (e), T stage (f), lymphatic metastasis (g), distant metastasis (h), and TNM stage (i) in pancreatic cancer; Figure S5: Sensitivity analysis of downregulated circRNAs for gender (a), differentiation (b), and lymphatic metastasis (c) in pancreatic cancer; Figure S6: Publication bias judged by Egger’s and Begg’s funnel plots of OS (a), DFS/PFS (b) for upregulated circRNAs and OS (c) for downregulated circRNAs in pancreatic cancer; Figure S7: Publication bias judged by Egger’s funnel plots of upregulated circRNAs for CA19-9 (a), nerve invasion (b), vascular invasion(c), differentiation (d), tumor size (e), T stage (f), lymphatic metastasis (g), distant metastasis (h), and TNM stage (i) in pancreatic cancer; Figure S8: Publication bias judged by Begg’s funnel plots of upregulated circRNAs for CA19-9 (a), nerve invasion (b), vascular invasion(c), differentiation (d), tumor size (e), T stage (f), lymphatic metastasis (g), distant metastasis (h), and TNM stage (i) in pancreatic cancer; Figure S9: Publication bias judged by Egger’s and Begg’s funnel plots of downregulated circRNAs for gender (a), differentiation (b), and lymphatic metastasis (c) in pancreatic cancer.
