# Supplementary material for: Prognostic, Diagnostic, and Clinicopathological Significance of Circular RNAs in Pancreatic Cancer: A Systematic Review and Meta-Analysis
Source: Cancers (Basel). 2022 Dec 14;14(24):6187. doi: 10.3390/cancers14246187 (PMC9777076; doi:10.3390/cancers14246187)
Supplement: Supplementary file 1 [file cancers-14-06187-s001.zip › supplementary 11132022/Table S2.docx]

**Table S2.** Assessment of the diagnostic accuracy and heterogeneity in subgroup analysis

| **Category** | **No. of studies** | **Sensitivity** | **Specificity** | **PLR^a^** | **NLR^b^** | **DOR^c^** | **I^2^ (%) of DOR** |
| --- | --- | --- | --- | --- | --- | --- | --- |
| Total | 12 | 0.79 (0.72-0.85) | 0.73 (0.66-0.79) | 2.91 (2.30-3.70) | 0.28 (0.21-0.39) | 10.22 (6.50-16.09) | 100.00 |
| **Year** |  |  |  |  |  |  |  |
| 2022 | 5 | 0.82 (0.78-0.86) | 0.73 (0.68-0.77) | 2.84 (1.88-4.29) | 0.26 (0.15-0.45) | 12.47 (5.17-30.07) | 80.8 |
| <2022 | 7 | 0.75 (0.70-0.79) | 0.71 (0.66-0.75) | 2.64 (1.97-3.55) | 0.34 (0.25-0.47) | 8.53 (4.97-14.65) | 59.1 |
| **Case size** |  |  |  |  |  |  |  |
| >60 | 6 | 0.79 (0.76-0.82) | 0.71 (0.67-0.75) | 2.66 (1.96-3.62) | 0.28 (0.18-0.45) | 10.43 (5.11-21.27) | 81.8 |
| ≤60 | 6 | 0.77 (0.71-0.82) | 0.73 (0.67-0.79) | 2.98 (1.94-4.58) | 0.33 (0.22-0.48) | 10.03 (4.98-20.20) | 59.7 |
| **Detected sample** |  |  |  |  |  |  |  |
| Blood | 5 | 0.74 (0.69-0.79) | 0.69 (0.63-0.74) | 2.33 (1.95-2.77) | 0.37 (0.24-0.56) | 7.45 (4.24-13.10) | 47.5 |
| Tissue | 7 | 0.81 (0.77-0.84) | 0.73 (0.69-0.77) | 3.18 (2.11-4.79) | 0.27 (0.19-0.38) | 12.66 (6.14-26.09) | 79.3 |

a, PLR: positive likelihood ratio; b, NLR: negative likelihood ratio; c, DOR: diagnostic odds ratio.
