# Supplementary material for: Prognostic, Diagnostic, and Clinicopathological Significance of Circular RNAs in Pancreatic Cancer: A Systematic Review and Meta-Analysis
Source: Cancers (Basel). 2022 Dec 14;14(24):6187. doi: 10.3390/cancers14246187 (PMC9777076; doi:10.3390/cancers14246187)
Supplement: Supplementary file 1 [file cancers-14-06187-s001.zip › supplementary 11132022/Table S3.docx]

**Table S3.** Results of univariate meta-regression analysis of diagnostic odds ratio.

| **Covariates** | ***p* value** | **RDOR** |
| --- | --- | --- |
| Year(≥2022/<2022) | 0.7132 | 1.30 (0.26-6.50) |
| Case size(>60/≤60) | 0.8271 | 0.86(0.17-4.27) |
| Detected sample(Blood/ Tissue) | 0.4889 | 0.65(0.16-2.66) |

RDOR: relative DOR.
