# Supplementary figures and images for: Prognostic, Diagnostic, and Clinicopathological Significance of Circular RNAs in Pancreatic Cancer: A Systematic Review and Meta-Analysis
Source: Cancers (Basel). 2022 Dec 14;14(24):6187. doi: 10.3390/cancers14246187 (PMC9777076; doi:10.3390/cancers14246187)

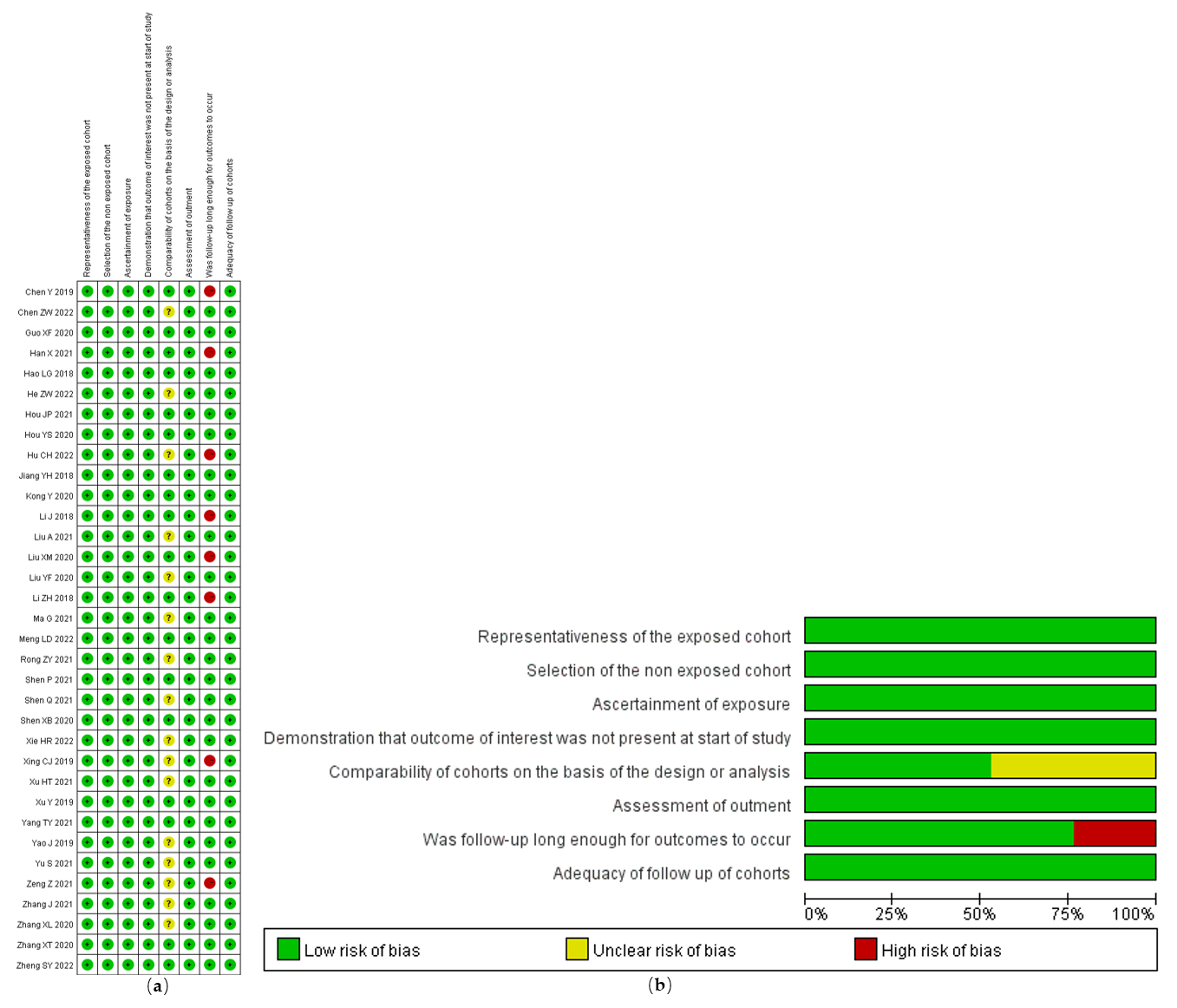

Supplement: Supplementary file 1 [file cancers-14-06187-s001.zip › supplementary 11132022/Figure S1.tif]

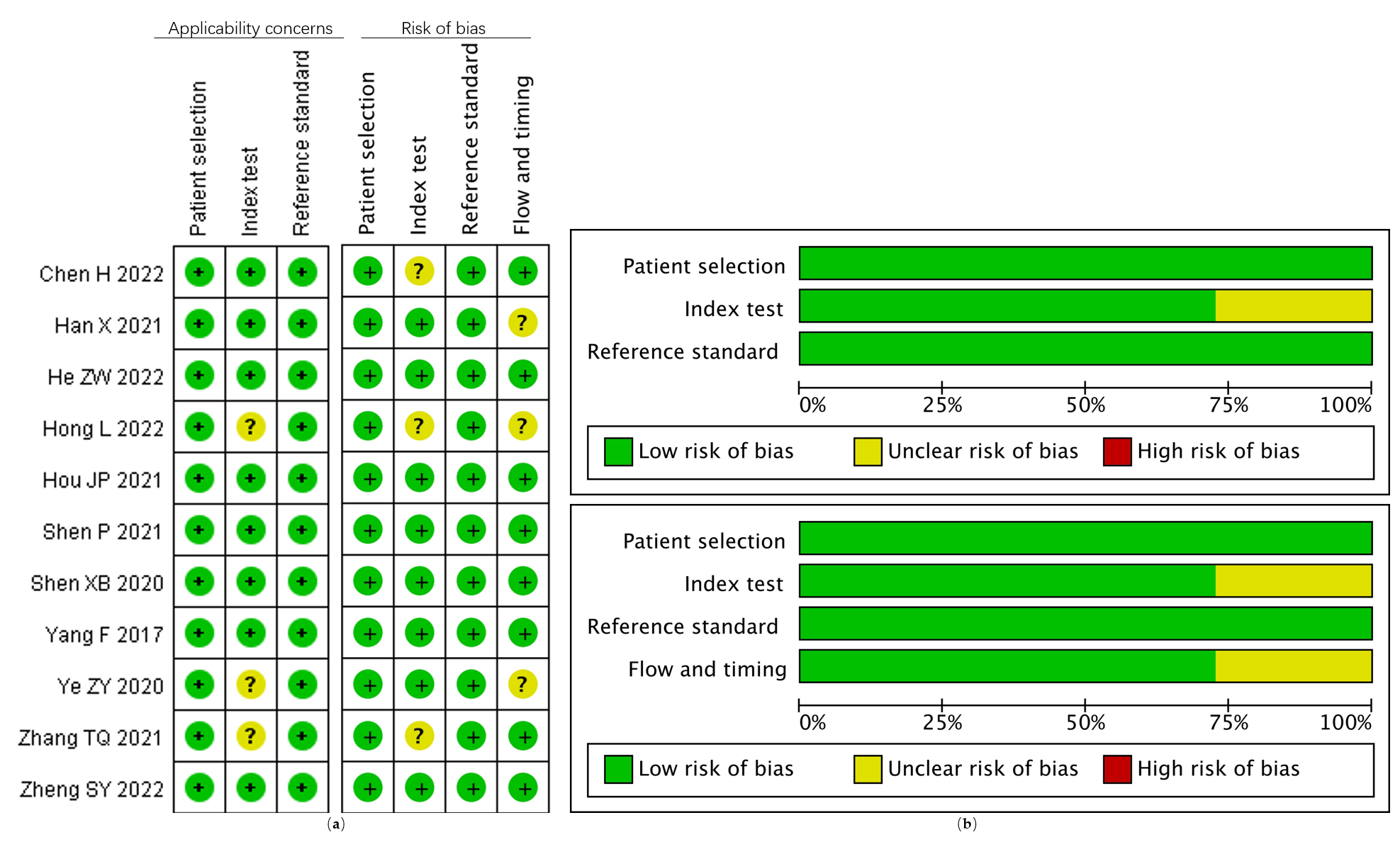

Supplement: Supplementary file 1 [file cancers-14-06187-s001.zip › supplementary 11132022/Figure S2.tif]

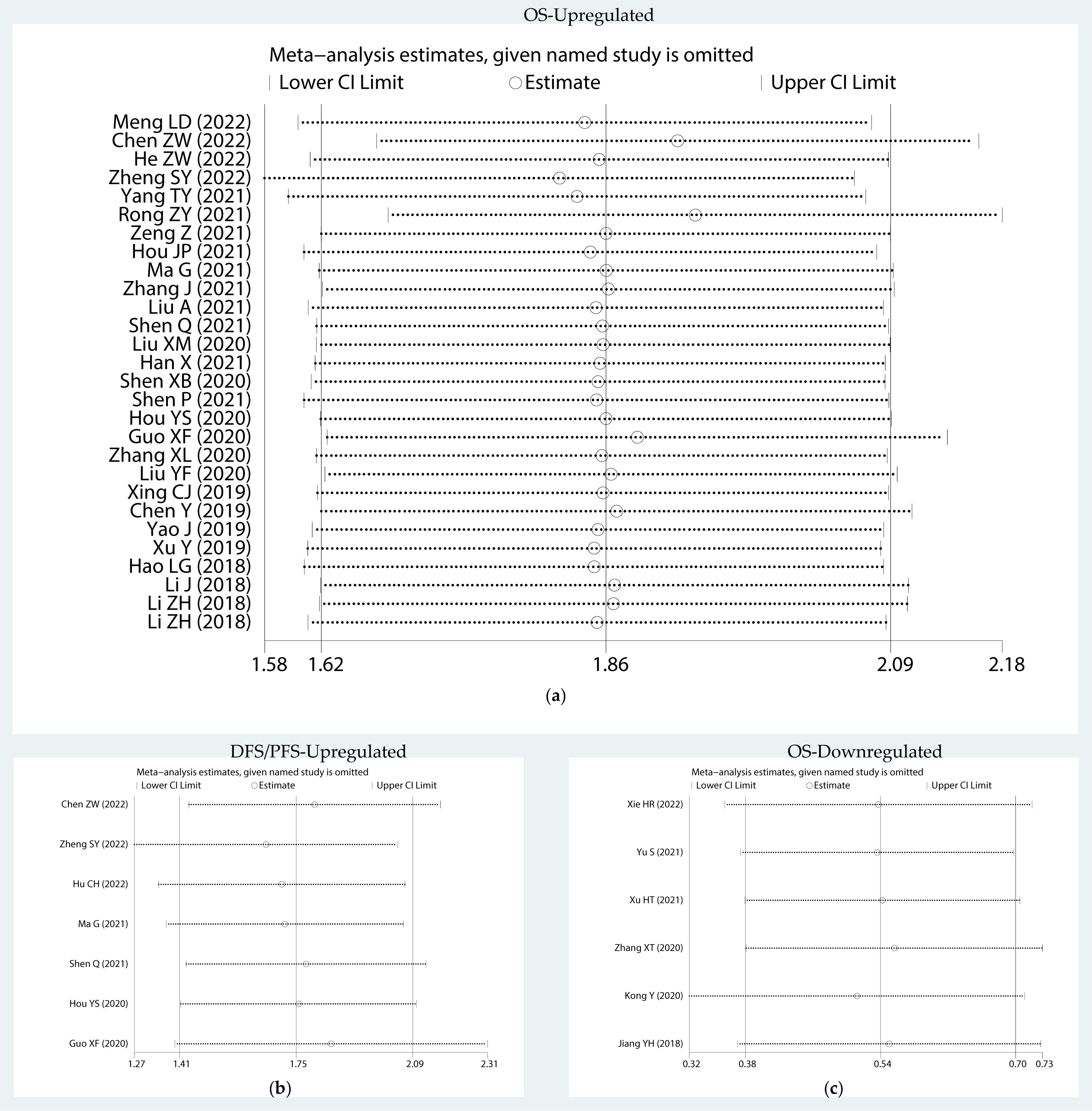

Supplement: Supplementary file 1 [file cancers-14-06187-s001.zip › supplementary 11132022/Figure S3.tif]

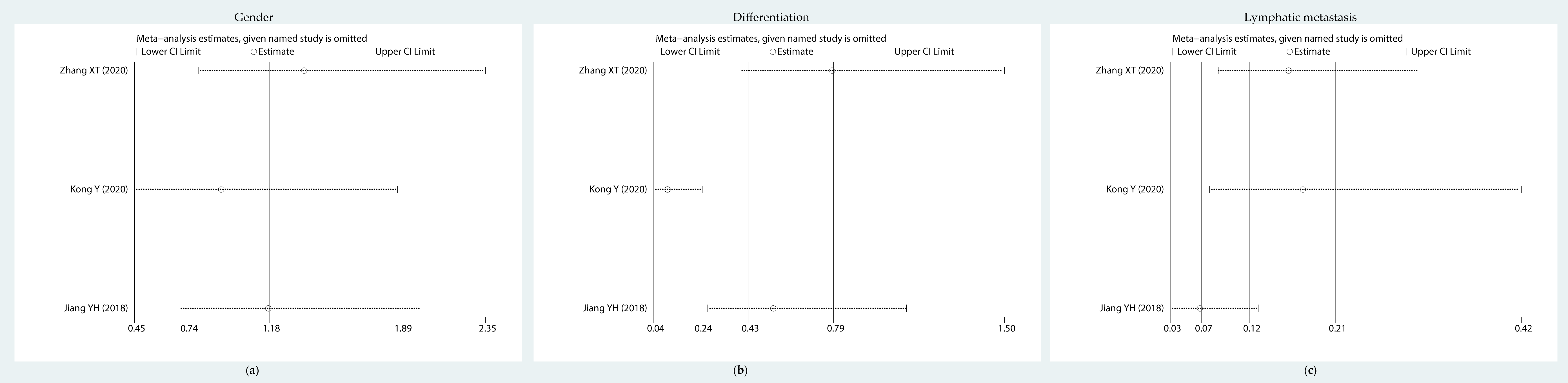

Supplement: Supplementary file 1 [file cancers-14-06187-s001.zip › supplementary 11132022/Figure S5.tif]

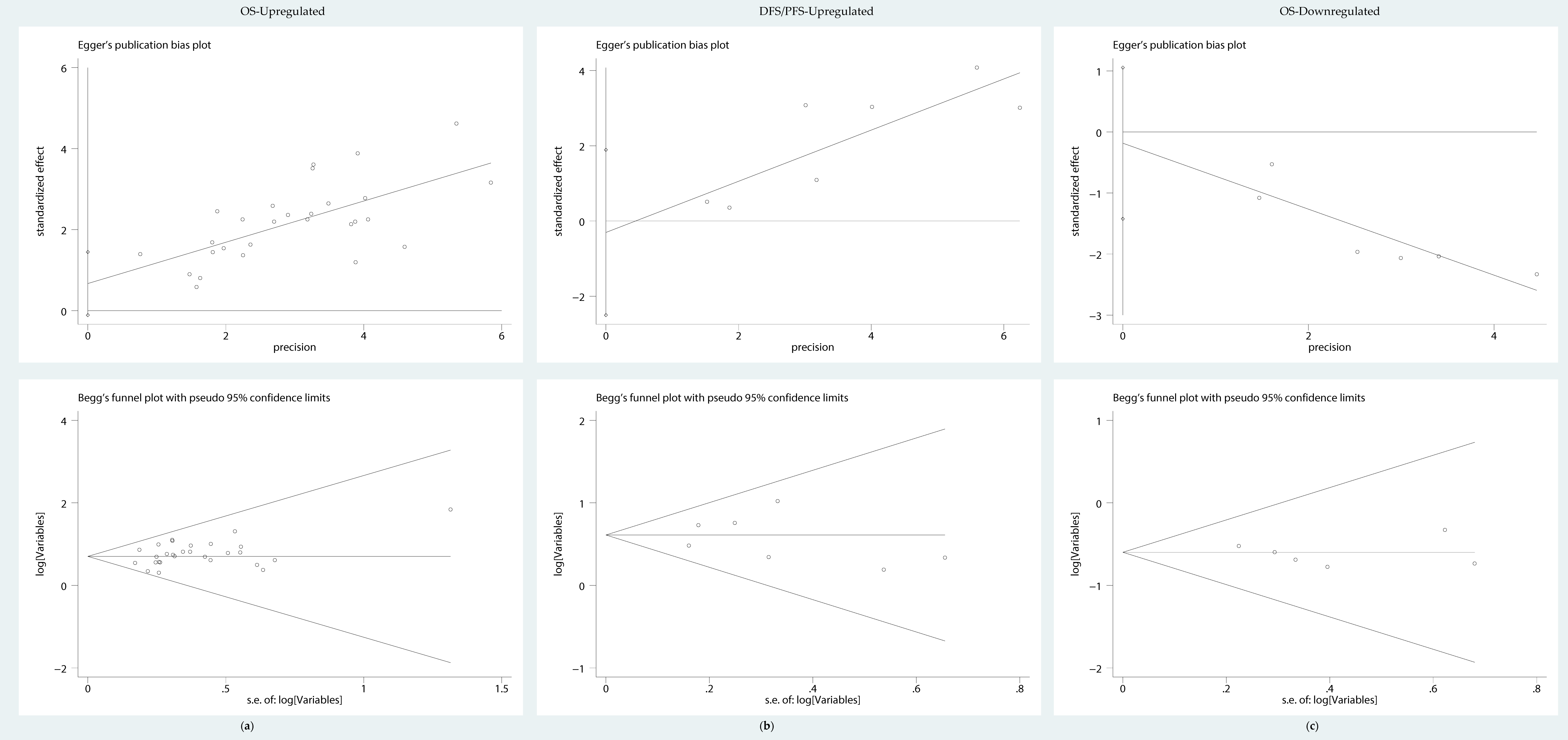

Supplement: Supplementary file 1 [file cancers-14-06187-s001.zip › supplementary 11132022/Figure S6.tif]

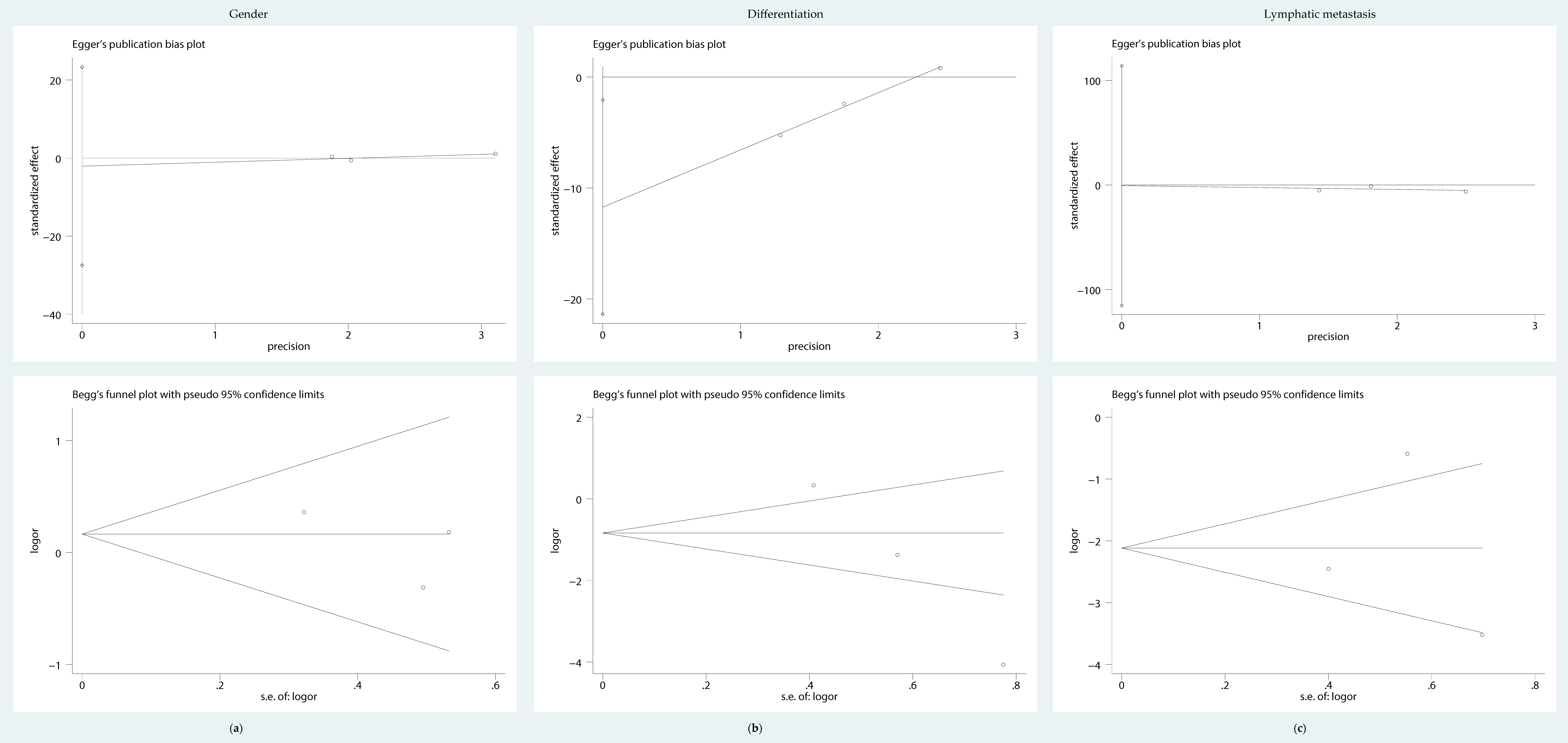

Supplement: Supplementary file 1 [file cancers-14-06187-s001.zip › supplementary 11132022/Figure S9.tif]
